# Analysis of *PLEKHS1* promoter mutation in preoperative thyroid nodule samples

**DOI:** 10.1002/cncy.70103

**Published:** 2026-05-04

**Authors:** Sarah Azad, Timothy E. Graham, Shiri Levy, Evana Velanzuela‐Sheker, David Bimston, Andrea Ferenczi, Yang Chen, Mohammed Alshalalfa, Yangyang Hao, Joshua P. Klopper, Richard T. Kloos, Anupam Kotwal

**Affiliations:** ^1^ Division of Diabetes, Endocrinology, and Metabolism University of Nebraska Medical Center Omaha Nebraska USA; ^2^ Diabetes & Endocrine Treatment Specialists Sandy Utah USA; ^3^ Henry Ford Health Detroit Michigan USA; ^4^ Memorial Healthcare System Hollywood Florida USA; ^5^ Endocrinology Associates, P.A. Scottsdale Arizona USA; ^6^ Veracyte, Inc. South San Francisco California USA

**Keywords:** thyroid nodules, mutations, genomics, RNA sequencing, thyroid cancer, Afirma

## Abstract

Indeterminate thyroid nodules carry a wide range of malignancy prevalence, necessitating incorporation of molecular testing to guide management. Molecular test results help guide prognosis to a certain extent, but additional prognostic factors need to be identified. Limited data have linked *PLEKHS1* promoter (*PLEKHS1p*) mutations with higher aggressiveness of thyroid cancer. Hence, we aimed to evaluate the diagnostic and prognostic value of *PLEKHS1p* mutations in preoperative thyroid nodules undergoing molecular testing. We assessed *PLEKHS1p* mutations in 9279 patient samples from April 2023 to June 2024 among indeterminate thyroid nodules ((B)ethesda III/IV) with Afirma GSC‐(S)uspicious results as well as among B V/VI cytology thyroid nodules. We also analyzed a subset of 20 consecutive cases positive for *PLEKHS1p* mutations with surgical resection for histology and for co‐occurring molecular alterations from the Afirma testing. *PLEKHS1p* mutations were positive in 60/9279 (0.6%) of patient samples with three times higher frequency in B VI compared to B III cytology nodules (1.36% vs 0.47%, *p* < .01). Among the 60 samples harboring *PLEKHS1p* mutations, 28 had the C593T hotspot mutation, 38 the G590A mutation, and six samples had both; four samples had concomitant *TERTp* mutations and 18 samples had concomitant *BRAFp.V600E* alterations. Our study demonstrated an overall low frequency of *PLEKHS1p* mutations, but this frequency was highest among malignant (B VI) cytology thyroid nodules. The frequency of *PLEKHS1p* mutations did not strongly correlate with the severity of thyroid cancer but the surgical sample size was limited. Further research is needed to clarify the role of *PLEKHS1p* mutations in thyroid nodules and cancer.

## Abstract 2

*PLEKHS1* promoter mutation is expressed at a low frequency in indeterminate but higher in malignant thyroid nodules.

## INTRODUCTION

Thyroid cancer is the most common endocrine malignancy, and its incidence has tripled over the past 30 years.^1^ Most diagnoses of thyroid cancer are in the setting of a single or multiple thyroid nodules with more than 60% of the population having these by the seventh or eighth decade of life.^2^ Approximately 20% of thyroid fine‐needle aspirates result as indeterminate thyroid nodules (ITNs), classified as (B)ethesda III or IV, where the risk of malignancy ranges from 6 to 40%.^3^ Molecular testing has been utilized to reduce the risk of unnecessary surgeries for indeterminate thyroid nodules.^4^ More recently, molecular tests have been leveraged for prognostic information, which can then influence the management not only for molecular suspicious ITNs (B III/IV), but also for cytologically suspicious for or malignant (B V/VI) thyroid nodules. These include assessment of late‐hit promoter mutations that increase the aggressiveness of thyroid cancers, include *TERT* promoter *(TERTp), TP53*, and genes in the *PI3K/AKT/mTOR* pathway, such as *PIK3CA* and *AKT1*.^5^ Although *TERTp* mutations are helpful in PTC prognostication, they occur in a small fraction of patients.^6^ Hence, there has been a search for additional prognostic factors, among which *Pleckstrin homology domain containing S1* (*PLEKHS1*) is a poorly characterized factor, whose promoter mutations have been identified in human malignancies, especially those where *TERTp* mutations are frequently observed.^7^, ^8^

**FIGURE 1:**
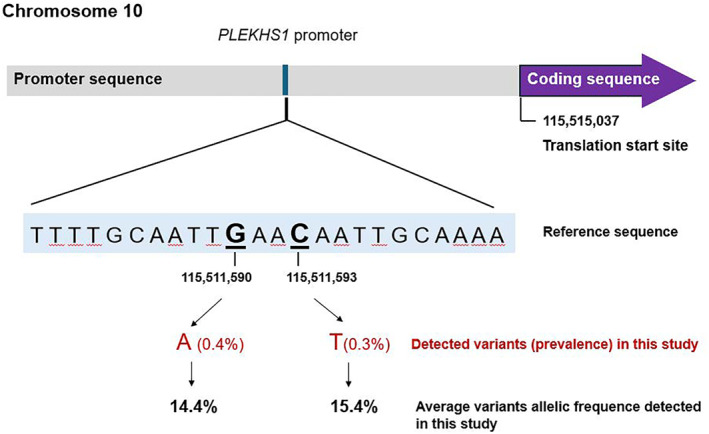
Genomic position and sequence of Pleckstrin Homology Domain Containing S1 (*PLEKHS1*) promoter mutations. Mutation coordinates are based on GRCh37/hg19.

The *PLEKHS1* gene encodes a pleckstrin homology domain relevant to several cellular processes including intracellular signaling and cytoskeletal organization. This gene is located on chromosome 10q25.3, encoding the *PLEKHS1* protein. Mutations affecting this gene are single‐nucleotide substitutions in the *PLEKHS1* proximal promoter (in intron 1 with a noncoding exon 1 from NM_024889.4): substitutions that effect guanine at position Chr.10: 115,511,590 and cytosine at position Chr.10: 115,511,593 from the GRCh37 (hg19) genomic coordinate (or c.‐20+70G and c.‐20+73C from NM_024889.4).^7^ These mutations are close to the translation start site of the *PLEKHS1* gene (−3447 and −3444 bp upstream of the translation start site) and are flanked by stretches of 10 bp on both sides that are palindromic to each other.^7^ Genomic position and sequence of *PLEKHS1* promoter (*PLEKHS1p*) mutations are illustrated in Figure 1.

In thyroid cancer, one study demonstrated that *PLEKHS1p* mutation was associated with a higher likelihood of lymph node and distant metastases, as well as shorter overall and disease‐free survival independent of *TERTp* presence.^9^ The underlying pathophysiology was shown to be associated with enhanced AKT phosphorylation and invasiveness. Another study demonstrated that *PLEKHS1p* mutation was associated with higher risk of radioactive iodine (RAI)–refractoriness in thyroid cancer independent of the *TERTp* presence.^10^ Although these studies demonstrate the association *PLEKHS1p* mutations with more aggressive thyroid cancer features, the diagnostic and prognostic significance of these mutations in thyroid nodules remains unknown. The aim of this study was to evaluate the diagnostic and prognostic value of *PLEKHS1p* mutations in preoperative thyroid nodules undergoing molecular testing.

## MATERIALS AND METHODS

In March 2023, the Afirma Genomic Resource for Intelligent Discovery research use only database was made available for investigators and includes the detection of the C593T and G590A *PLEKHS1p* hotspot mutations.^11^ The database is a research tool to advance the science of thyroid nodule molecular diagnostic and prognostic markers. The *PLEKHS1p* mutation was assessed as part of the *TERTp* mutation DNA assay, which is an opt‐in request on the Afirma GSC test requisition form.^12^, ^13^ The PLEKHS1p mutation detection was performed using the same workflow described in the Afirma *TERT* assay analytical verification paper.^12^ Specifically, genomic DNA was extracted from thyroid nodules using the Qiagen AllPrep Micro Kit and prepared for amplicon library construction using the AmpliSeq Library PLUS (384 reactions) with a custom AmpliSeq DNA panel, followed by sequencing on the Illumina MiniSeq Sequencing System (System Suite v2.2.1). Raw sequencing data were processed using Illumina Local Run Manager v2.4.1.2636 with the DNA Amplicon module v2.1.0.19 for quality control, alignment of sequencing reads to the hg19 reference genome to generate BAM files, and variant calling to generate variant call format files. Mutations were called using the following criteria: read depth ≥500, variant allele frequency >3%, and variant quality ≥30. Analytical sensitivity was assessed during assay development using positive control materials and demonstrated 100% detection at ≥5% variant allele frequency with a 10 ng DNA input.

We assessed *PLEKHS1p* mutations in 9279 patient samples from April 2023 to June 2024 among ITNs (B III/IV) with Afirma GSC‐(S)uspicious results as well as among B V/VI cytology thyroid nodules where the Afirma Xpression Atlas and the *TERTp* analysis were ordered as part of routine clinical care at the discretion of the ordering clinician. This was to evaluate demographic data in *PLEKHS1p*+ cases and evaluate the frequency of concordant *BRAFp.V600E* and *TERTp* mutations.

We also analyzed a subset of consecutive cases positive for at least one of the targeted *PLEKHS1* promoter mutations with surgical histology (*n* = 20) for co‐occurring expressed molecular alterations from the Afirma Xpression Atlas^14^ and pathology outcomes, under an institutional review board (IRB)–approved protocol (WCG IRB #DHF 005‐077). The 20 cases selected were those that were made available to the investigators on request under the IRB‐approved study.

Finally, chi‐square test was used for assessing the statistical association between PLEKHS1p mutation status and other variables.

## RESULTS

**FIGURE 2:**
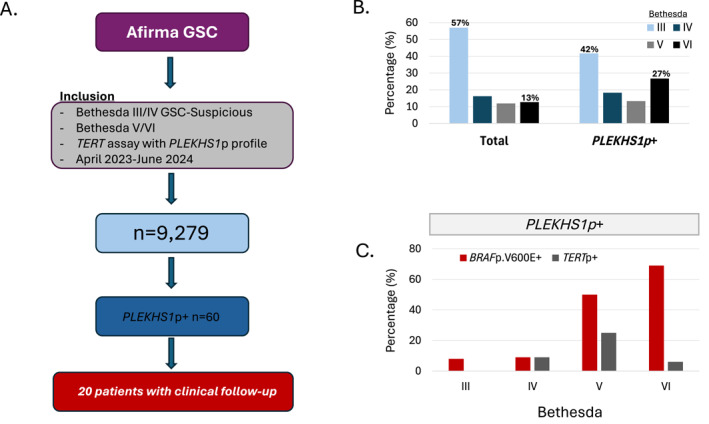
(A) Flow diagram of study samples and the number of samples assessed positive for *PLEKHS1p*. (B) The proportion of samples overall and those with *PLEKHS1p* by Bethesda category as shown in Table 1. (C) The proportion of PLEKHS1p+ samples with BRAFp.V600E and TERTp co‐mutations as shown in Table 2. *The B III and B IV nodules reported were GSC‐S.

*PLEKHS1p* mutations were assessed in 9279 patient samples (Figure 2A). Seventy‐four percent were from females and 57% were B III, 16% B IV, 12% B V, and 13% B VI. The median age, sex, Bethesda category, and number of *BRAFp.*V600E‐positive samples in the whole cohort are shown in Table 1. Even though age was trending to be higher in *PLEKHS1p+* group, the difference was not statistically significant (*p* = .41). The prevalence of B VI was higher in the *PLEKHS1p*+ samples compared to the whole cohort (26.7% vs 12.7%). The frequency of *PLEKHS1p* mutations was nearly three times higher in nodules with B VI cytology compared to B III cytology (16/1177 [1.36%] vs 25/5,293 [0.47%], chi square *p* < .01) (Table 1 and Figure 2B).

**TABLE 1:** Clinical and genomic characteristics of the whole cohort (*n* = 9279) and the subset of nodules that are *PLEKHS1p*+; the proportions of sex, Bethesda groups, *BRAF p.V600E*, and *TERT* promoter mutations were assessed.

Variable	Total (*n* = 9279)	*PLEKHS1p*+ (*n* = 60)
Age (years) median [IQR]	54 [40‐67]	58.1 [43‐67.1]
Sex		
Male	2448 (26.4)	12 (20%)
Female	6829 (73.6%)	48 (80%)
Bethesda group		
Afirma GSC suspicious III	5293 (57%)	25 (41.7%)
Afirma GSC suspicious IV	1507 (16.2%)	11 (18.3%)
V	1108 (11.9%)	8 (13.3%)
VI	1177 (12.7%)	16 (26.7%)
*BRA p.V600E*+	1912 (20.6%)	18 (30.0%)
*TERTp*+	492 (5.3%)	4 (6.7%)
*PLEKHS1p*+*	60 (0.6%)	60 (100%)
C593T	28 (0.3%)	28 (46.7%)
G590A	38 (0.4%)	38 (63.3%)

*Six patients had both PLEKHS1p mutations.

*PLEKHS1p* mutations were positive in 60/9279 (0.6%) of patient samples. The proportions of each hotspot mutation (C593T and G590A) assessed, and concomitant positive *TERTp* mutations and *BRAFp.*V600E mutations are shown in Table 2 and Figure 2C.

**TABLE 2:** The number of each hotspot mutations among the 60 *PLKEHS1*+ samples; the concomitant positive *TERT*p mutations and *BRAFp.V600E* mutations are shown.

Bethesda group	Total	*BRAF* p.V600E+	*TERTp*+	*PLEKHS1*p. C593T+	*PLEKHS1*p. G590A+	*PLEKHS1*p. C593T+ and G590A
GSC‐S III	25	2	0	11	14	0
GSC‐S IV	11	1	1	7	7	3
V	8	4	2	3	7	2
VI	16	11	1	7	10	1

Among the 60 patient samples harboring *PLEKHS1p* mutations, 28 had the C593T hotspot mutation, 38 the G590A mutation, and six samples had both. Additionally, four samples had concomitant *TERTp* mutations and 18 samples had concomitant *BRAFp.*V600E alterations. Among the six samples with both *PLEKHS1p* mutations, two were BV and both had *TERT* C228T+ and *BRAFp.V600E*+, and one BVI was *BRAFp.V600E*+.

Table 3 shows the case findings for 20 nodules with *PLEKHS1p* mutations where surgical histology was available: 15 from GSC‐S ITNs (B III/IV) and five from B V/VI. Among this subset, 40% (6/15) of ITNs were malignant on surgical histology and met 2015 American Thyroid Association criteria for low‐risk cancer, of which five of six (83%) had coalterations: *KRAS* p.G12D, *DICER*1 p.E1705K, *NRAS* p.Q61R + *PIK3CA* p.E545K + *TERT* p.C228T, *NRAS* p.Q61R, and *FGFR2::VCL* fusion.

**TABLE 3:** Characteristics of the 20 nodules with *PLEKHS1* promoter mutations where surgical histology was available; age ranges are provided to minimize identification in this cohort of patients with the rare PLEKHS1 promoter mutation.

Nodule size (cm)	Sex	Age range (y)	Bethesda	*PLEKHS1*p.C593T	*PLEKHS1*p.G590A	XA + *TERTp* result	Surgery type	Tumor type	Tumor size (cm)	AJCC Stage	ATA Risk
4.1	F	55–60	III	−	+	−	TT	FA	4.3	−	−
2.8	F	40–45	IV	+	−	*KRAS* p.G12D	RL	FVPTC with oncocytic features	3.5	T2NxMx	Low
1.9	M	65–70	III	+	−	−	LL + isthmusectomy	Adenomatoid hyperplasia	1	−	−
2.1	F	60–65	III	+	−	*DICER1* p.E1705K	Isthmusectomy	miFTC	2	T1bNxMx	Low
2.4	M	55–60	III	+	−	−	LL + Isthmusectomy	FA with oncocytic change	2.1	−	−
1.2	M	30–35	III	−	+	*PAX8*::*GLIS3*	LL	Hyalinizing trabecular tumor	0.9	−	−
2.6	F	45–50	III	−	+	*NRAS* p.Q61R	TT	FH	2	−	−
4.2	F	65–70	IV	+	−	−	RL	MiOTC	4.3	T3aN0aMx	Low
1.8	F	75–80	IV	+	+	*NRAS* p.Q61R, *PIK3CA*:p.E545K, *TERT* p.C228T	TT	PTC	0.6	T1aN0aMx	Low
0.69	F	45–50	IV	−	+	*NRAS* p.Q61R	TT	PTC	0.2	T1aN0aMx	Low
2.9	F	40–45	IV	+	+	*FGFR2*::VCL	TT	PTC (Warthin‐like subtype), Infiltrative	3	T2NxMx	Low
3.2	F	70–75	III	−	+	−	LL	FA	3.2	−	−
6.6	F	65–70	III	−	+	−	LL	FA	4.8	−	−
1.2	F	55–60	III	−	+	−	TT	FA	0.2	−	−
4.7	F	65–70	III	−	+	−	LL + Isthmusectomy	ON	4.5	−	−
1.4	F	60–65	VI	−	+	*BRAF* p.V600E, *DICER1* p.Q7R	TT + BLND	PTC	1.5	T1bN0aMx	Low
2.8	F	65–70	VI	+	−	*BRAF* p.V600E	TT + CND + LND	PTC	4.7	T3bN1bMx	High
1	F	55–60	V	−	+	*NTRK3*::QSTM1	RL	IFVPTC	1.1	T1bNxMx	Low
2	F	85–90	V	−	+	*BRAF* p:V600E	TT + CND	PTC	2.6	T2N1aMx	High
3.95	F	25–30	VI	+	−	*NRAS* p.Q61R	TT	PTC	4.8	T3aN0aMx	Intermediate

Abbreviations: BLND, bilateral lateral neck dissection; CND, central neck dissection; FA, follicular adenoma; FTC, follicular thyroid cancer; FV, follicular variant; LL, left lobectomy; LND, lateral neck dissection; mi, minimally invasive; ON, oncocytic neoplasm; OTC, oncocytic thyroid cancer; PTC, papillary thyroid cancer; RL, right lobectomy; TT, total thyroidectomy.

Among this subset, two of nine (22%) of ITNs that were benign on surgical histology had coalterations (*PAX8::GLIS3* fusion and *NRAS* p.Q61R). Only one of eight (12.5%) ITNs with an isolated *PLEKHS1p* mutation was malignant on surgical histology (minimally invasive oncocytic carcinoma). All five B V/VI nodules were malignant on surgical histology and met American Thyroid Association criteria for high‐ (*n* = 2), intermediate‐ (*n* = 1), or low‐ (*n* = 2) risk thyroid cancer.

For 10 of 11 carcinomas where the mitotic rate was noted on the pathology report, none had a mitotic rate >4 mitosis per 2 mm^2^.

## DISCUSSION

Our study investigating a large sample of Afirma GSC suspicious B III/IV as well as B V/VI cytology thyroid nodules, demonstrated an overall low frequency of *PLEKHS1p* mutations but this frequency was highest among malignant (B VI) cytology thyroid nodules.

The demographic characteristics of the patient cohort are consistent with clinical practice, in that the median age was 54 years and females predominated. Most thyroid nodules had B III and IV cytology, as those more often undergo molecular testing for diagnostic assessment to guide the next step. The overall frequency of *TERTp* mutations in this cohort is consistent with prior reports, as ITNs (B III/IV) are less likely to harbor these mutations as compared to B V/VI nodules.^15^, ^16^ The *BRAFp.V600E* prevalence is slightly higher than would be seen in all molecularly tested nodules given the relatively higher proportion of B V/VI samples and exclusion of Afirma GSC Benign B III/IV samples from this cohort.

In our study, the frequency of *PLEKHS1p* mutations increased as the cytologic category progressed from B III through B VI. The three‐times‐higher frequency of *PLEKHS1p* mutations in nodules with B VI cytology compared to B III cytology demonstrates that the presence of this mutation is associated with more suspicious cytology diagnosis. However, this may not hold much clinical significance, as among the 20 samples with available surgical histology, the presence of *PLEKHS1p* mutations did not strongly correlate with the severity of thyroid cancer, even among the B V/VI samples. This suggests that preoperative knowledge of PLEKHS1p status in nodules that are diagnostic for malignancy would not alter management, though this is based on a very small sample size.

Two studies have assessed *PLEKHS1p* mutations in thyroid cancer, demonstrating its association with more aggressive features. Xing et al.^9^ conducted RNA‐sequencing among 93 patients with papillary thyroid cancer (PTC), and 18 patients with anaplastic thyroid cancer, as well as five thyroid cancer–derived cell lines. They found that *PLEKHS1p* was overexpressed in thyroid carcinomas including PTC, and its higher expression was associated with lymph node and distant metastases, and shorter overall and disease‐free survival among patients with PTC. *PLEKHS1p* overexpression also predicted shorter patient survival in PTCs lacking *TERTp* mutations. Cell line experiments showed that *PLEKHS1p* activation contributed to the increased activity of the AKT pathway, which promotes aggressiveness of thyroid cancer, and hence would be the likely pathophysiological basis for this association.

Jung et al.^10^ conducted targeted deep sequencing of 157 cancer‐related genes of 67 tumor tissues of primary thyroid tumors (*n* = 46) and distant metastatic tumors (*n* = 21) from the 61 patients. They found that *PLEKHS1p* mutations (*n* = 6, 10%) were more common in the RAI‐refractory cases and this was independent of *TERTp* mutation. Hence, this study demonstrated that *PLEKHS1p* mutation was associated with higher risk of RAI refractoriness of thyroid cancer, indicating a more aggressive phenotype. However, in independently collected PTCs without initial distant metastasis (*n* = 75), a *PLEKHS1p* mutation was only found in one case that developed distant metastasis during the follow‐up period.

Thyroid cancer cell line analyses support the possibility of a link with adverse clinical outcomes but also raise questions regarding the correlation between *PLEKHS1p* mutations and the mRNA expression in thyroid tumors. One such study by Landa et al.^17^ analyzed 30 thyroid cancer cell lines and found that two cell lines harbored clonal heterozygous *PLEKHS1p* mutations, and three additional cell lines maintained subclonal *PLEKHS1p* mutations. However, overall *PLEKHS1* mRNA levels in cell lines were low and homogeneous, suggesting that there may be a lack of correlation of the *PLEKHS1p* mutations and the mRNA expression in primary thyroid tumors.

Despite these prior reports^9^, ^10^ linking *PLEKHS1* promoter mutations with aggressive behavior and RAI‐refractory disease, several biological and methodological factors may explain why our study did not identify a strong prognostic signal in preoperative nodules. One possibility is stage‐dependent acquisition, where *PLEKHS1p* mutations represent a late molecular event that becomes more prevalent only after progression to advanced or metastatic disease; such mutations may therefore be underrepresented in earlier‐stage nodules sampled preoperatively, such as in our study. Additionally, clonal heterogeneity may limit the prognostic value of *PLEKHS1p* status in fine‐needle aspiration samples, as subclonal promoter mutations may not be sufficiently represented to drive phenotypes in early lesions. Another explanation is the distinction between promoter mutation status and gene expression. Prior studies often reported associations based on *PLEKHS1* overexpression rather than promoter sequence variants; promoter mutations in isolation may not necessarily translate into transcriptional activation in thyroid tumors, consistent with data showing minimal correlation between *PLEKHS1p* mutation and mRNA levels in cell lines.^17^ Finally, the phenotypic impact of *PLEKHS1p* mutations may depend on interaction with other mutations, such as *TERTp* or *BRAF*p.V600E. In our cohort, few *PLEKHS1p*‐positive nodules harbored *TERTp* comutations, and only a subset carried *BRAF*p.V600E, potentially attenuating any independent prognostic effect. Taken together, these factors may explain the discrepancy between previous associations with aggressive disease and the lacking prognostic signal observed in this preoperative, predominantly early‐stage thyroid nodule cohort.

Our study has limitations, mostly pertaining to the limited number of samples where surgical histology was also available as well as the lack of long‐term follow up on outcome; therefore, our study is not powered to assess the prognostic significance of *PLEKHS1p* mutations in thyroid cancer. However, our study’s strengths include that it is the first such study investigating the frequency and correlation of *PLEKHS1p* mutations with thyroid nodule cytology and surgical histology. The large sample size, enriched B III and B IV with suspicious Afirma GSC, but also including B V and B VI nodules is a strength, although the exclusion of ITNs that were Afirma GSC‐Benign may limit this study’s ability to account for all thyroid nodules that harbor the *PLEKHS1p* mutation. Therefore, it is possible that the overall frequency of *PLEKHS1p* mutations is even lower than reported here among thyroid nodules that are not diagnosed with B II cytology. However, we would expect a very small proportion of Afirma GSC‐B nodules to harbor *PLEKHS1p* mutations, and its prevalence among B V and B VI nodules would not be affected by GSC‐B. Another strength is the targeted assessment of *PLEKHS1p* mutation status, identifying specific hotspots that are mutated, and evaluating concomitant driver and promoter alterations that contribute to thyroid carcinogenesis. Availability of surgical histology in a subset of the cohort allowed us to correlate cytology diagnosis with the gold‐standard surgical histology for definitive diagnosis among samples with *PLEKHS1p* mutations.

To conclude, this targeted sequencing analysis of a large cohort of thyroid nodules demonstrates that *PLEKHS1p* mutations are rare in thyroid nodules undergoing molecular testing and are present more frequently in B VI compared to ITNs. In contrast with data in metastatic thyroid cancer, isolated *PLEKHS1p* mutations detected in ITNs do not seem to predict a higher risk of cancer nor aggressive histology as compared with other Afirma GSC suspicious thyroid nodules, though this is a qualified assessment based on the small sample size in this study. Based on this limited data set, detection of a PLEKHS1p mutation in a thyroid lesion should not initiate more aggressive management than would otherwise be undertaken given clinical, ultrasound, and other validated molecular findings. Further studies are needed to clarify the role of *PLEKHS1p* mutations in thyroid nodules and thyroid cancer to harness the full potential of molecular information for improving patient care.

## Author Contributions

**Sarah Azad:** Data curation; writing—original draft; and writing—review and editing. **Timothy E. Graham:** Methodology and writing—review and editing. **Shiri Levy:** Data curation; methodology; and writing—review and editing. **Evana Velanzuela‐Sheker:** Methodology; data curation; and writing—review and editing. **David Bimston:** Methodology; data curation; and writing—review and editing. **Andrea Ferenczi:** Methodology; data curation; and writing—review and editing. **Yang Chen:** Methodology; data curation; and writing—review and editing. **Mohammed Alshalalfa:** Investigation; formal analysis; methodology; and writing—review and editing. **Yangyang Hao:** Formal analysis; methodology; and writing—review and editing. **Joshua P. Klopper:** Conceptualization; methodology; writing —review and editing; and writing—original draft; investigation. **Richard T. Kloos:** Conceptualization; methodology; writing—original draft; writing—review and editing; and investigation. **Anupam Kotwal:** Investigation; conceptualization; methodology; writing—review and editing; writing—original draft; and project administration.

## CONFLICT OF INTEREST STATEMENT

Anupam Kotwal has received consultancy honoraria from Veracyte, Inc. Richard T. Kloos and Yang Yang Hao are Veracyte equity owners and prior employees. Yang Chen, Mohammed Alshalalfa, and Joshua P. Klopper are employees and equity owners of Veracyte, Inc.
